# The label-feedback effect is influenced by target category in visual search

**DOI:** 10.1371/journal.pone.0306736

**Published:** 2024-08-01

**Authors:** Paolo Frugarello, Elena Rusconi, Remo Job

**Affiliations:** 1 Department of Psychology and Cognitive Science, University of Trento, Rovereto (Trento), Italy; 2 Centre of Security and Crime Sciences, University of Trento – University of Verona, Trento, Italy; Education University of Hong Kong, HONG KONG

## Abstract

The label-feedback hypothesis states that language can modulate visual processing. In particular, hearing or reading aloud target names (labels) speeds up performance in visual search tasks by facilitating target detection and such advantage is often measured against a condition where the target name is shown visually (i.e. via the same modality as the search task). The current study conceptually complements and expands previous investigations. The effect of a multimodal label presentation (i.e., an audio+visual, AV, priming label) in a visual search task is compared to that of a multimodal (i.e. white noise+visual, NV, label) and two unimodal (i.e. audio, A, label or visual, V, label) control conditions. The name of a category (i.e. a label at the superordinate level) is used as a cue, instead of the more commonly used target name (a basic level label), with targets belonging to one of three categories: garments, improper weapons, and proper weapons. These categories vary for their structure, improper weapons being an ad hoc category (i.e. context-dependent), unlike proper weapons and garments. The preregistered analysis shows an overall facilitation of visual search performance in the AV condition compared to the NV condition, confirming that the label-feedback effect may not be explained away by the effects of multimodal stimulation only and that it extends to superordinate labels. Moreover, exploratory analyses show that such facilitation is driven by the garments and proper weapons categories, rather than improper weapons. Thus, the superordinate label-feedback effect is modulated by the structural properties of a category. These findings are consistent with the idea that the AV condition prompts an "up-regulation" of the label, a requirement for enhancing the label’s beneficial effects, but not when the label refers to an ad hoc category. They also highlight the peculiar status of the category of improper weapons and set it apart from that of proper weapons.

## 1 Introduction

The idea that language and visual perception may reciprocally interact [1], whereby visual perceptual experiences shape language processing [2, 3] and language, in turn, influences perceptual representation [4–6], is not new [7, 8]. The interweaving of visual perceptual processes and language has intrigued researchers for decades and has been widely studied from opposing perspectives [9, 10]. However, its precise nature remains unclear and no consensus has been reached so far on the degree to, and the mechanisms through, which perceptual processes may be influenced by language [11, 12]. The reported effects of language on visual perception can be broadly distinguished in online and offline effects [5]. Our study is concerned with the online effects of language on a visual search task.

In visual search and detection tasks, the provision of a linguistic expectation for the incoming perceptual input is a common manipulation used to demonstrate that language can influence visual perception [5]. Object names (that is, basic level labels) have been widely used to induce such linguistic expectation and their influence on visual perception has been accounted for by postulating that they can pre-activate visual features that distinguish a to-be-detected object from a non-target [5, 13–15]. Further, top-down augmentation of perceptual representations can be up-regulated by activating the label to a greater degree [16]. In this regard, the label-feedback hypothesis [16, 17] proposes that hearing [14, 15, 18–22] or reading aloud [17, 22, 23] the visually presented name of a target object may facilitate performance in a following detection trial more than silently reading a visually presented label [17, 22]. It is proposed that a label could influence early visual processing by priming linguistic and perceptual representations associated with a concept, as the activated lexemes dynamically interact with feature detectors [23]. By establishing a linguistic expectation within which the incoming input is processed, the word label may thus produce a label-feedback effect [23] or label advantage effect [15] due to its aid in the formulation of perceptual hypotheses on the incoming visual input [24].

A purely visual presentation of the label activates object-diagnostic features [16, 17], and the addition of a concomitant auditory presentation may boost visual search [16], via a mechanism of multimodal facilitation [17, 23]. Indeed, when two independent sensory inputs (e.g. an auditory and a visual input) interact by providing consistent information, their effectiveness may be higher than when the same information is provided in a single sensory modality [17].

Overall, the enhancement linked to the auditorily presented label has been related to higher sensitivity at detecting a visual target [18, 19], shorter response times (RTs) at picture verification [15], visual search [17, 22, 23] and detection tasks [18, 20, 21, 23], and higher accuracy at visual search [17, 22, 23] and recognition tasks [21], compared to a range of control conditions, such as: silent reading [17, 22] and/or visual cue [19, 22], no valid [18, 23] or absent cue [18, 20, 23], nonverbal sound [14, 15, 21]. The effect of the additional oral label on perception tends to be transient, lasting a few hundreds of milliseconds after label onset [16], and would be maximal when the target is high in imagery concordance [17, 22] (i.e. the degree of agreement between the depicted object and its mental image) [25, 26].

In most of the studies conducted thus far on the label-feedback hypothesis, labels have been systematically presented at basic level, corresponding to the name of the target object (e.g. *guitar*), while labels at superordinate level, related to the category of the target (e.g. *musical instruments*), have never been employed. Labels at superordinate level were introduced in other contexts, such as in a task of visual recognition of indeterminate two-tone Mooney images [27]. They did yield a significant facilitation, which was not, however, as strong as that provided by the basic-level label (a nearly 4-fold increment vs. a 16-fold increase in accuracy in recognizing Mooney images relative to a free-naming baseline) [27]. In the literature on visual search, the superordinate label has been used once, to test for the presence of a basic-level label superiority effect [28]. Labels were presented only visually prior to the onset of a search grid and superordinate labels were compared to pictorial previews, subordinate labels and basic level labels. The data indicated that, besides an overall advantage of pictorial cues, label specificity may facilitate visual search in the guidance to target phase (i.e. from the onset of the search display to the first fixation of the target), whereas a basic-level label superiority effect unfolds at the verification stage (i.e. from the first fixation of the target to response selection) [28]. This might provide general support for predictive processing accounts of visual perception, positing that prior knowledge (provided by labels at different hierarchical levels) may feedback to modulate lower levels of perceptual processing [29–31]. However, performance after a superordinate level label ranked last, indicating that the potential for application of findings on label facilitation to performance critical real-world search tasks may not be as straightforward as one may wish [23]. In aviation security screening [32], superordinate categories are more useful as a guide to routine searches than basic or subordinate categories (for example, a screener may be typically instructed to look for *weapons* rather than for a *handgun* or a *Beretta 92*), whereas a narrow focus on the latter may be detrimental. Therefore, also from a translational point of view it is important to establish whether and for what type of categories a putative superordinate label feedback effect may facilitate performance in visual search tasks.

In the current study, we investigated the effect of superordinate labels and of their presentation modality on performance in a visual search task where participants are required to assess whether a collection of objects contains an object belonging to the superordinate category, in the fastest and most accurate way.

The name of a category (i.e. a label at the superordinate level in the categorical hierarchy, e.g. *garment*) was used as a cue instead of the more commonly used target name (a basic level label, e.g. *shirt*), in order to have an insight into the type of features activated by the label. From a theoretical standpoint, the label feedback hypothesis assumes that the effects of labels on visual search stem from the preactivation of features associated to the concept the label refers to. However, not all members of a superordinate category share the same visual features, or they may share very few of them. In this case, the activated visual features may be very general, e.g. allowing to distinguish *birds* from *furniture*, or they may be those characterizing most, and the most typical, members of the category [see, e.g. 33, 34]. The use of superordinate category labels may thus allow us to ascertain whether they provide sufficient linguistic expectations to be instrumental in the formulation of perceptual hypotheses on the incoming visual input, based on general (as opposed to specific) visual knowledge [21] and affordances [26, 35].

Crucially, we presented the label auditorily and visually (audio+visual label, AV, the *target* condition), and compared this condition to another multimodal audiovisual condition, i.e. a visually presented target category name plus white noise (white noise+visual label, NV, the multi-modal control condition). This permitted us to avoid operationalizing the label-feedback effect as a difference between a multimodal and a unimodal condition, a methodological issue which both Cho [22] and Hebert et al. [23] have already noticed in the previous literature [17], while offering evidence with a novel control condition for the label-feedback account. A white noise control condition has been previously used in the context of multimodal interaction studies as an auditory stimulus unrelated to the pictures of an event scene in the search display [36] or in a recognition task [37, 38], to be contrasted either to a sound congruent with the target [36, 37], for example the sound of glass shattering for “break”, or to no sound (the target conditions) [38], or a sound congruent with a distractor [36, 37]. With respect to the white noise control condition, the mean response time was shorter for the target-related sound condition (indicating auditory enhancement of the search) and longer for the distractor-related sound condition [36, 37]. Additionally, the naming of pictures that show an object with a typical sound was delayed by white noise [38]. Since white noise does not provide any relevant linguistic information, in addition to being a valid baseline, the condition with white noise may help rule out that the auditory stimulus in the multimodal label condition acts as an attention-capture device rather than a feature activation trigger, as posited by the label feedback hypothesis.

Our primary hypothesis was preregistered and concerned specifically the contrast between the AV experimental condition and the NV control condition. We predicted an overall effect on reaction times (RTs) (accuracy shows a ceiling effect almost in every study mentioned above, [17–23] for correct responses which can be formulated as follows:

$$\text{Mean}\;\text{correct}\;\text{RTs}\;\left(\text{AV}\;\text{superordinate}\;\text{label}\right)<\text{Mean}\;\text{correct}\;\text{RTs}\;\left(\text{NV}\;\text{superordinate}\;\text{label}\right).$$

All the other variables were introduced and measured or manipulated for exploratory purposes.

In order to capture potential changes in sensitivity, in addition to RTs and accuracy, we measured performance also in terms of dprime (a measure derived from Signal Detection, Theory) [39, 40], considered an index of early perceptual processing [41, 42]. Our participants were required to respond whether the target is present or absent, as in Lupyan & Ward [18] and Lupyan & Spivey [19], where the oral label-feedback effect is shown to increase sensitivity in detecting a visual target compared to absent [18] or visual cue [19]. To assess whether multimodal presentation confers an advantage [43–47] when a superordinate label is used, the label was also presented in a unimodal format, either auditorily (audio label, A; unisensory auditory control condition) or visually (visual label, V; unisensory visual control condition).

To assess whether the superordinate label-feedback effect may be modulated by category type and have relevant implications for real-world searches, three different target categories were included. Two of them (proper weapons and garments) refer to well-defined standard categories, whereas the third (improper weapons) refers to a set of items whose primary function is different than that implied by their label. We recently documented the psychological reality of improper weapons [48, 49], which include everyday tools that can be used as weapons or carried with the purpose to injure. The definition of improper weapons is derived from the classification of weapons applied within the Italian Penal System (in accordance with international standards; e.g., for the United Kingdom, see the Offensive Weapons Act and Criminal Justice Act; for the United States, see the Federal Switchblade Act and United States Code, Title 18) and includes, for example, sharp and blunt objects (e.g. knives, hammers; art. 4 of Italian law n.110, April 18, 1975). We found that improper weapons are distinguished (albeit with blurry lines) from both proper weapons (i.e. created with the purpose of causing harm) and everyday objects that do not have the intrinsic characteristic of causing harm (e.g. books, spoons) by a series of attributes (dangerousness, frequency of incidents in which these objects are involved, feeling of control, and familiarity), suggesting that it would be worth to consider improper weapons as a psychologically “autonomous” category [48, 49]. Previous research on language-cued visual search considered neither this specific category nor ad hoc categories at large. Garments, but not weapons, have been extensively used in other studies with basic-level category labels [14, 15, 17, 18, 20–23]. Given the differences in intrinsic properties [48, 49] between proper and improper weapons, and between weapons and garments, we considered it particularly informative to evaluate each of the three categories separately, for an additional characterization of the reported effects.

Finally, previous research suggests that anxiety may impair attentional control, while increasing attention to specific types of stimuli, such as those associated with threats [50–52]. Angry facial expressions [53, 54], dangerous animals [55, 56], weapons [55] or objects that can be used this way [55], can more readily be detected than matched non-threatening stimuli. This phenomenon is referred to in the literature as the threat superiority effect (TSE) [57]. A perceiver-based influence that is thought to play an important role in threat-based visual search tasks is the level of trait anxiety. For example, a positive correlation has been reported between the level of trait anxiety and RTs for angry face targets [54, 58], and in patients with schizophrenia for non-social threats, such as snakes [59].

Therefore, before the start of the experimental session, participants were individually assessed for trait anxiety, in order to probe its potential relation with performance depending on the target category and interact with the label-feedback effect (see Methods, 2.1.1 Individual assessment).

To summarize, the study provides original data on four related aspects: the label-feedback effect operationalized as an advantage for audiovisual over white-noise+visual labels in visual search; the effectiveness of superordinate level category labels in igniting the label-feedback effect; whether a label-feedback effect is found with an ad-hoc category referring to a secondary function of items (that is, improper weapons); the susceptibility of search performance and of the label-feedback effect to trait anxiety.

The study was preregistered before the start of any data collection and the preregistration file can be viewed on the Open Science Framework [60] at the following link:

https://osf.io/c8y5v/?view_only=1dd9cc2356444294a4ccd4bf80ae0a05.

## 2. Methods

The Methods follow closely our preregistered plan [60], with one exception. In order to prevent participants from depending solely on auditory information to perform the task, due to faster early processing times of spoken compared to visual words [61], the audio label or white noise have been provided 200 milliseconds after the onset of the visual label (see 2.3 Procedure). As a result, both in the multimodal and in the unimodal conditions, the visual label lasted 1700 ms, while the auditory label (or white noise) lasted 1500 ms.

### 2.1 Participants

As per our preregistration plan, we obtained full data from 200 male and female Italian speaking, adult (over 18 years of age) participants, able to express their informed consent. The target number was reached after recruiting 283 volunteers (of them, 83 either finished the study too quickly or too slowly, based on pre-established criteria; see 2.4.1 Data exclusion and missing data).

They all reported normal cognitive functions and no central and/or peripheral visual or hearing problems. The Prolific platform was used to recruit participants. Prior to the experiment, all participants provided online written informed consent and, after completing it, they were paid € 4.06 each.

The sample size was determined on the basis of an a priori power analysis calibrated on the main hypothesis of the experiment (see Introduction), with the use of the software program G*Power 3.1 [62]. We set an objective of .90 power to detect a small effect size of *d* = .25 at the standard .02 alpha error probability with a paired-samples Student’s t-test. This would require a sample size of 181 (one-tailed) but we placed our target at 200 participants, to increase the probability that the desired minimum number of participants was available after potential exclusions at the data analysis stage (i.e. after completion of data collection; see 2.4.1 Data exclusion and missing data). The study was approved by the University of Trento Human Research Ethics Committee on 4^th^ September 2020 (Protocol 2020–019). The whole procedure was realized in accordance with the Helsinki Declaration (World Medical Association, 2013). Recruitment took place from 22/08/2021 to 25/08/2021.

#### 2.1.1 Individual assessment

Given the presence in this study of categories of objects that could be defined as threats, individual differences in a self-reported trait (trait anxiety) were measured via the *STAI-Y State Trait Anxiety Inventory (Y2)* [63] before the beginning of the experimental session.

### 2.2 Stimuli

#### 2.2.1 Labels

Three labels were employed, one for each target category: “proper weapon” (in Italian: “arma propria”), “improper weapon” (in Italian: “arma impropria”), “garment” (in Italian: “indumento”). The labels for the two weapon categories were cast from the Italian Penal System. Regarding the visual label format, Helvetica, font size 18, black text was displayed on a white background for the visual label. The audio format for each of the three labels were recordings of a male voice saying aloud the name of the category generated via Audacity software with an intensity of 70 dB SPL.

#### 2.2.2 Target

For each of the three categories, i.e. proper weapons, improper weapons, garments, ten target objects were chosen, which were a selection of the items included in Frugarello et al. [48]. Measures of imagery concordance [19, 22], familiarity and visual complexity for these targets (see S1 Table for details) were available from Frugarello et al. [49], where an independent but comparable sample of participants answered the following questions [25]: "How much does the image you see match your idea of the object?", “How familiar is this object, according to how usual or unusual the object is in your experience?” and “How complex is this object in terms of the amount of detail or intricacy of lines?” respectively, with a 5-point scale ranging from 1 (Not at all/Very simple) to 5 (Extremely/Very complex) [25]. Three one-way ANOVAs (familywise significance criterion: p_FW_ < .02, lowered to p < .007 per individual ANOVA after Bonferroni correction) showed that the three categories do not differ in terms of imagery concordance (*p* = 0.44), and visual complexity (*p* = 0.09). The three categories differed in familiarity (*p* < .001), in the expected direction [49]: proper weapons < improper weapons < garments (all *p*s < .001).

The following items were included in our study:

- *Garments*: undershirt, jeans, sneaker, shirt, sock, hat, jacket, cap, skirt, heel shoe.
- *Improper weapons*: baseball bat, cutter, screwdriver, hammer, scissors, cooking thermometer, tube, razor, blowpipe, pliers.
- *Proper weapons*: puncher, dynamite, gun, bullets, bomb, rifle, flare gun, dagger, baton, detonator.

In the visual search display, the target and the distractors were placed on an invisible 5x5 search grid, which was centered on the display and comprised 25 equidistant virtual positions [17, 22]. Both targets and distractors were standardized to occupy 10% width × 15% height of the screen displayed on a 17-inch Dell monitor at a resolution of 1280 × 1024.

OsWeb, the online runtime for OpenSesame experiments, a JavaScript library that interprets and performs OpenSesame experiments, adapted the size of the search grid and stimuli to the size of the computer screen of the participants (further information available here: https://osdoc.cogsci.nl/manual/osweb/osweb/#about-osweb).

The target appeared in half of the trials and was absent in the other half, where it was replaced by a distractor. When present, the target was displayed along with 11 distractors; when absent, 12 distractors were displayed. Targets were positioned pseudo-randomly to ensure even frequencies of appearance across the four quadrants of the search grid. Each target appeared four times, once in each Label format block. Each block consisted of 60 trials (30 with target present, 30 with target absent), and each trial contained a single target. As such, the total number of trials was 240: 10 targets x 3 categories x 4 label formats (AV, NV, A, V) x 2 target status (present/absent).

#### 2.2.3 Distractors

The distractors were selected from a variety of databases providing color photographs of real-world objects on a white background. Living organisms (animals and plants), food, and items with large physical dimensions (i.e. items that could not fit into a suitcase, such as vehicles, furniture, buildings etc.) were not considered. Each distractor appeared four times during the experiment, just as each target. As a result, 330 distractors were required for the 120 trials in which the target was present, and 360 distractors were required for the 120 trials in which the target was absent. At the start of the experiment, another 23 distractors were used for practice trials (see 2.3 Procedure). The 713 distractors required for the study (690 for the experimental session and 23 for the practice session) were sourced from the following databases:

368 (out of 1468) from Brodeur, Guérard & Bouras [64].
103 (out of 551) from Ni et al. [65].
55 (out of 360) from Moreno-Martínez & Montoro [66].
39 (out of 106) from Martínez, Matute & Goikoetxea [67].
117 (out of 450) from Saryazdi et al. [68] (2018).
31 (out of 174) from Viggiano, Vannucci & Righi [69].

#### 2.2.4 Search displays

For each search display (trial-level) we calculated eleven standard visual complexity indices: contrast, correlation, energy, homogeneity, compression ratio [70], colourfulness, number of colors, entropy, edge density [71], feature congestion [72] and subband entropy [72]. The "graycoprops" function of MATLAB (R2017b) was used to calculate the first four indices, which measure the attributes of the Grey Level Co-occurrence Matrix (GLCM) [73]. The fifth through tenth indices were generated in R (4.0.3) using the packages "imager" (0.42.3), "imagefluency" (0.2.3), "EBImage" (4.32.0), and "jpeg" (0.1–8.1). The latter two were calculated using MATLAB (R2017b) [72].

With a familywise significance criterion of p_FW_ < .02 (lowered to .0018 per individual test after Bonferroni correction), eleven one-way ANOVAs showed that search displays did not differ on each of these complexity measures across the three categories (all *p*s > .05). The values of the indices for each trial are reported in S1 File.

### 2.3 Procedure

The experiment had a maximum duration of 30 minutes and was carried out in an online mode, combining OSWeb (OpenSesame Web, 3.3.8) [74] with JATOS [75]. At the beginning of the experiment, the participants completed the STAI-Y (Y-2). Then an introductory phase followed, in which a brief definition of the categories to be searched was displayed (e.g. “Any object whose main purpose is to offend the individual is considered a proper weapon. It was made specifically as an instrument for offence and for no other reason”), along with examples of objects that belong to each category. For each category, ten objects similar but not visually identical to the targets (that is, they were different instances of each target) were displayed, one by one. Moreover, the participant was informed that the label formats were in auditory and/or visual mode, and examples of audio stimuli similar but not identical to those used in the experimental session (in this introductory phase, a nonword and a white noise with different frequency and bandwidth but with the same duration as the experimental stimuli) were given. Both white noises, the one used in the introductory phase and the one used in the experimental task (monotrack, 44.100 Hz sampling frequency with 32-bit float, bandwidth 43 Hz-20,005 kHz) [76], were generated via Audacity software. The initial intensity of the audio files was set to 70 dB SPL [76], however participants were given the option to change it during the practice phase if the sound/white noise were too loud or too weak. At the end of the introductory phase, the participant received instructions on how to successfully complete the task and was assigned a response rule (association of responses “yes” and “no” to response keys Z and M on a QWERTY keyboard, counterbalanced between participants).

The main task—requiring participants to decide whether a target object was present in the displayed array of objects—was divided in four main blocks, each characterized by a different label format.

At the beginning of each block, experimental trials were preceded by a practice session with twelve trials, whose targets were taken from the examples of objects belonging to each of the categories shown in the introductory phase; thus, targets in practice trials were visually different from those in experimental trials. In each trial (practice and experimental), visual labels were presented for 1.7 seconds, followed by a search display after a 500 milliseconds fixation point.

**Fig 1:**
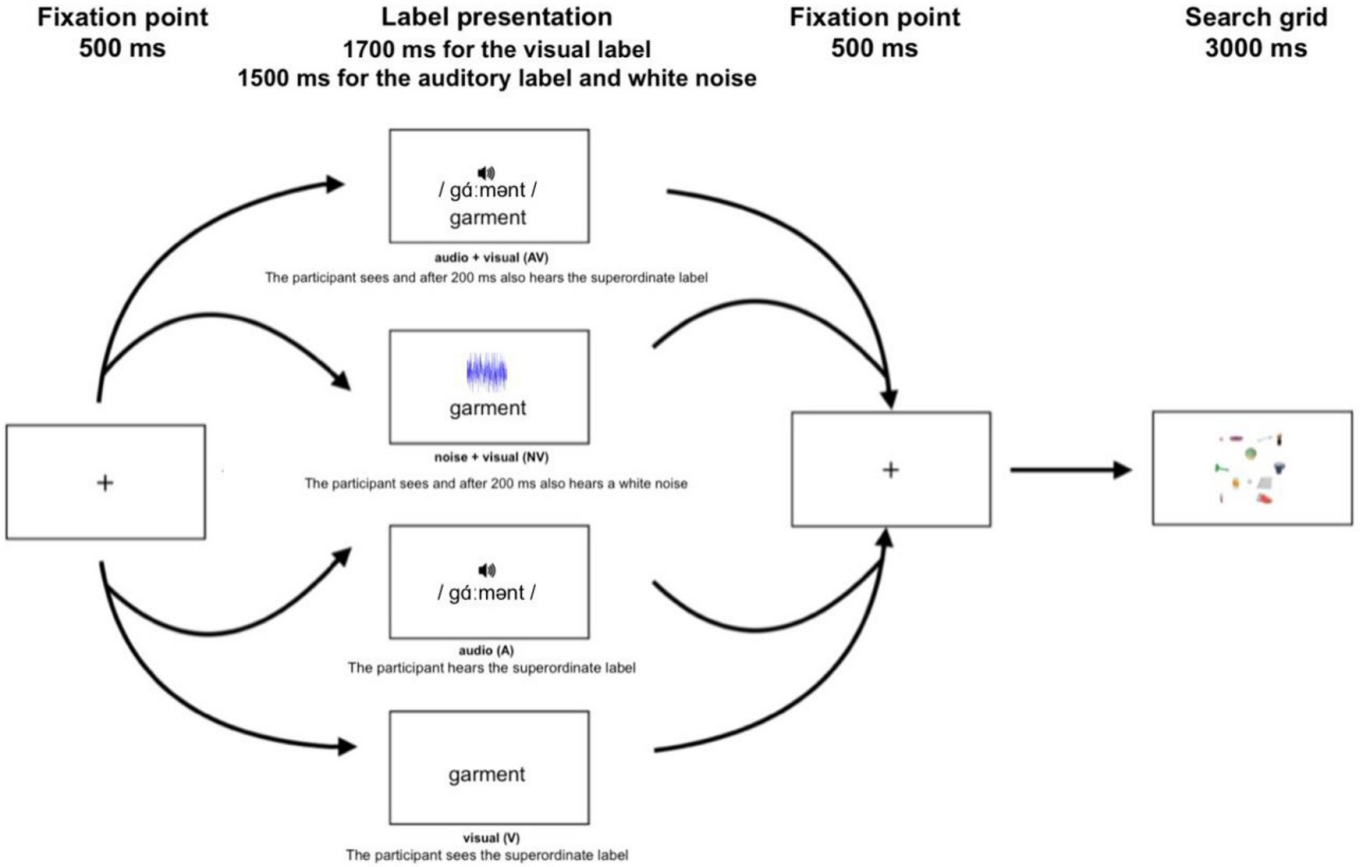
Trial structure for the experimental task. Labels are always presented in advance of the search display; targets are present in half of the trials.

Audio labels and white noise were presented for 1.5 seconds (with onset 200 ms after the visual label in the AV and NV conditions), allowing the participant to process both labels without disregarding one of the two [77–79]. Each search display contained twelve objects and remained on the screen for a maximum time of 3 seconds, which was also the deadline for response (Fig 1).

In practice trials, a feedback on accuracy was provided immediately after a response, with targets, when present, highlighted with a red circle on the image after the feedback. In each experimental trial (with or without response), only feedback on cumulative accuracy for the current block was shown. After the feedback, a fixation point appeared, signaling the start of the next trial. The inter trial interval (i.e. ITI) varied randomly between 250 and 1000 ms.

The order of appearance of the blocks was counterbalanced across participants. Within each block, trials appeared randomly, with the constraints that no more than two targets from the same category could appear in a row and no more than three identical responses were requested in a row.

### 2.4 Data analysis

#### 2.4.1 Data exclusion and missing data

As established at the preregistration stage [60], on reaching 200 participants, a quality control of the data was performed. Participants who completed the experiment in a time-frame that was 3 standard deviations (SD) below or above the average of all available participants were excluded and replaced until the target sample size was reached again and another check was performed. Once the target sample size was reached, data collection was closed.

Participants with 50% accuracy or less were then excluded from the analysis, as were participants with more than 5% missing data [80], or who had less than 5% missing data but all relating to trials of the same label format or relating to the same category (this is referred to the number of empty cells out of a total of 60 for each label format,10 targets x 3 categories x 2 target status, or 80 cells for each category, 10 targets x 4 label formats x 2 target status).

Correct RTs faster than 200 ms, and trials with late response, hence not logged within the permitted time frame (3 seconds), or missing responses were excluded from the analysis [81] and considered as missing data [60].

The final number of participants whose data were analyzed was 186.

#### 2.4.2 Statistical analyses

The variables considered are the following: RTs in AV and NV label format (confirmatory analyses), RTs in each label format for each category (exploratory analyses), dprime for each label format for each category (exploratory analyses), RTs for each category and STAI-Y2 scores (exploratory analyses).

Confirmatory analyses were performed using R (4.0.3) on correct RTs to experimental trials after trimming outliers by the modified recursive procedure of Van Selst & Jolicoeur [82] with a threshold of 3.5 SD, in accordance with the preregistered protocol (i.e. t-test between RTs_AV_ and RTs_NV_).

The dataset was then submitted to a series of exploratory analyses using the “Ez” (4–4.0), “Psychreport” (3.0.1), “Effectsize” (0.4.5), “Cocor” (1.1–4) [83] R’s packages.

In exploratory analyses, we computed robust estimate intervals and performed hypothesis testing using the bootstrap approach (2000 repeats) for all crucial contrasts, in case some of the data did not meet all of the assumptions of parametric analyses [84]. The wBoot package was used for robust paired t-tests and for post-hoc comparisons following exploratory ANOVAs [85]. Outputs from standard statistical tests have been reported together with CIs of the robust test. p values from robust tests have been detailed where they differ in significance from standard testing. Parametric tests were used for investigation of the association between trait-anxiety and performance.

In order to assess whether correct RTs differed across the factors Label format and Category, a 4 × 3 repeated measures ANOVA was conducted, with Label format (AV, NV, A, V) and Category (proper weapons, improper weapons, garments) as within-participant factors. Only target-present trials were included.

In order to assess whether dprime differed across the factors Label format and Category, a 4 × 3 repeated measures ANOVA was conducted, with Label format (AV, NV, A, V) and Category (proper weapons, improper weapons, garments) as within-participant factors.

A family-wise significance criterion of p_FW_ < .02 was used (lowered to p < .01 per individual ANOVA after Bonferroni correction). In case of significant interactions, pairwise comparisons were conducted with Bonferroni’s post-hoc test.

For the *STAI-Y (Y2)*, Cronbach’s alpha was calculated. An exploratory analysis was performed to assess whether trait anxiety was linked to the participant’s performance.

A Pearson’s correlation was used to assess the relation between RTs on correct trials between each category and STAI-Y2 scores. The family-wise alpha threshold was p_FW_ < .02 (lowered to p < .006 per individual test after Bonferroni correction). Comparisons between the correlation indices were then performed with the Hittner’s test [86]. A family-wise significance criterion of p_FW_ < .02 was used (lowered to p < .006 per each comparison after Bonferroni correction).

## 3. Results

After reaching the total of 200 participants (106 males, age range = 18–73 years, average age = 27.5 years) set in our preregistration plan, eight participants were discarded because their accuracy was equal or lower than 50%, and another 6 participants because their missing data were > 5%. The final sample available for analyses is thus of 186 participants (101 males; age range = 18–64 years, average age = 27.6 years). After removing outlier trials (0.73% of total trials) using the van Selst and Jolicoeur’s method [82] and incorrect responses (8% of total trials), the preregistered analysis was conducted (see 3.1 Pre-registered analysis), followed by a series of exploratory analyses (see 3.2 Exploratory analyses). On average, our final sample of participants responded accurately to 87.5% (sd = 0.4) of the trials, with a response time of 1405 ms (sd = 530). In line with the literature [87], RTs were slower in target absent trials (M = 1712 ms, sd = 480) than in target present trials (M = 1099 ms, sd = 400), and accuracy was lower in target present (M = 83.7%, sd = 0.55) than in target absent trials (M = 91.4%, sd = 0.61). A small positive correlation between RTs and accuracy was thus found in overall performance (*ρ*_(185)_ = .06; *p* < .001). When considering target absent and target present trials separately, a speed-accuracy tradeoff was found for target absent trials (*ρ*_(185)_ = .16; *p* = .001), but not for target present trials (*ρ*_(185)_ = .02; *p* = .61). After testing for and confirming the presence of a label-feedback effect across all trials (as per preregistered analysis), our following analyses on RTs were mainly focused on target present trials, that is the condition driving the significant label-feedback effect across all trials (see below).

### 3.1 Pre-registered analysis

A significant difference was found in correct RTs between the AV label condition and the NV label condition t(185) = -3.72, *p* = 0.008, Bootstrapped: M = -39.21, 98% CI [(-13.40)]. On average, RTs were 39 ms faster in the AV label condition (M = 1207.7 ms, sd = 129.8) than in the NV label condition (M = 1246.5 ms, sd = 126.2).

### 3.2 Exploratory analyses

#### 3.2.1 RTs

Two separate follow-up t-tests were performed to assess whether the advantage of the AV label condition over the NV label condition held for both target absent and target present trials. In target absent trials, no significant difference was found between the AV label condition (M = 1389.3 ms, sd = 141.3) and the NV label condition (M = 1411.7 ms, sd = 167.5) [t(185) = -1.05, *p* = 0.14, Bootstrapped: M = -22.12, 98% CI (21.12)]. In target present trials, RTs significantly differed between the AV label condition and the NV label condition [t(185) = -6.55, *p* < .001, d = .24, Bootstrapped: M = -55.04, 98% CI (-40.77)]. On average, RTs were 55 ms faster in the AV label (M = 1026.11 ms, sd = 221.78) than in the NV label condition (M = 1081.30 ms, sd = 237.77) for target present trials. The presence of a label-feedback effect was also examined for accuracy (percentage of correct responses), although this was not the variable of primary interest, by comparing AV and NV overall, in trials with the target present, and in trials with the target absent. The same pattern was found as in RTs, with a significant advantage for the AV over the NV condition across trials [t(185) = 4.28, *p* < .001; M_AV_ = 90.6%, sd_AV_ = 0.56; M_NV_ = 88.5%, sd_NV_ = 0.65] and in trials with the target present [t(185) = 5.27, *p* < .001; M_AV_ = 88.4%, sd_AV_ = 0.69; M_NV_ = 85.1%, sd_NV_ = 0.71] but not in trials with the target absent [t(185) = 1.47, *p* = .07; M_AV_ = 92.8%, sd_AV_ = 0.77; M_NV_ = 91.8%, sd_NV_ = 0.95]. Therefore, only trials with the target present were included in the subsequent exploratory ANOVA for RTs.

**Fig 2:**
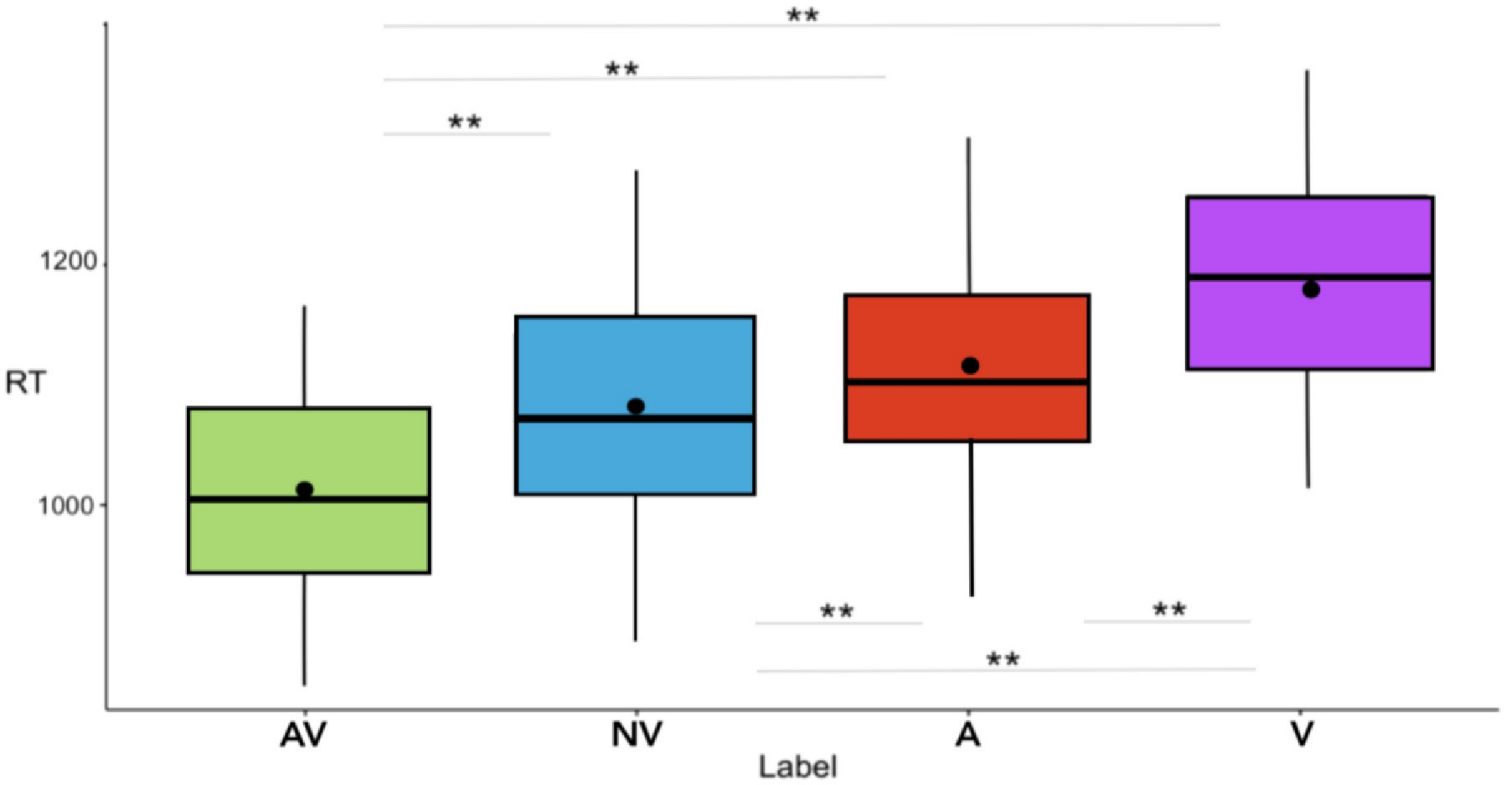
Mean RTs (y-axis) following each of the four label formats (x-axis) are shown. AV = audio + visual; NV = noise + visual, A = audio, V = visual. The significance of the pairwise comparisons between label formats (Bonferroni’s post-hoc tests) is indicated by asterisks: ** (p < .001). The box’s range, including whiskers, corresponds to the mean +/-3*SD, the thick line running the width of the box represents the median, the point inside the box represents the mean.

The 4 (Label format) × 3 (Category) repeated measures ANOVA revealed main effects of Label format [F(3, 555) = 82.78, MSE = 51307.65, *p* < .001, η_p_^2^ = .31] and Category [F(2, 370) = 597.88, *p* < .001, MSE = 52524.79, η_p_^2^ = .76]. RTs after the AV label were 55 ms, 98 ms and 145 ms shorter than after the NV label [t(1115) = -7.71, *p* < .001, d = .24, Bootstrapped: M = -55.23, 98% CI (-71.88, -38.29)], the A label [t(1115) = -12.33 *p* < .001, d = .38, Bootstrapped: M = -98.39, 98% CI (-115.8, -81.33)], and the V label [t(1115) = -17.24, *p* < .001, d = .57, Bootstrapped: M = -145.17, 98% CI (-164, -126.5)], respectively (mean RTs are reported in Table 1 for each Label format; statistical significance has been assessed via Bonferroni post-hoc tests). Moreover, RTs were 43 ms and 89 ms shorter following the NV label than the A label [t(1115) = -4.98, *p* = .006, d = .16, Bootstrapped: M = -42.92, 98% CI (-62.86, -22.54)] and the V label [t(1115) = -10.21, *p* < .001, d = .34, Bootstrapped: M = -89.96, 98% CI (-110.6, -69.65)], respectively. Finally, RTs were 47 ms shorter following the A label than the V label [t(1115) = -4.87, *p* < .001, d = .16, Bootstrapped: M = -46.40, 98% CI (-68.05, -25.16)] (see Fig 2).

**Table 1:** Mean RT for each condition of label format are reported below.

**Label format**	**Mean RT (sd)**
Audio + visual (AV)	1026.11 (221.78)
Noise + visual (NV)	1081.30 (237.77)
Audio (A)	1124.51 (288.34)
Visual (V)	1171.22 (281.04)

With respect to Category, Bonferroni’s post-hoc tests revealed that RTs for proper weapons (M = 1225 ms, sd = 217.55) were 89 ms and 284 ms slower than for improper weapons (M = 1136.19 ms, sd = 189.10) [t(1457.7) = 8.44, *p* < .001, d = .35, Bootstrapped: M = 89.23, 98% CI (70.72, 106.7)] and garments (M = 940.68 ms, sd = 170.79) [t(1406.7) = 28.08, *p* < .001, d = .9, Bootstrapped: M = 284.81, 98% CI (264.4, 305.6)], respectively. RTs were 195 ms slower for improper weapons in respect to RTs for garments [t(1470.8) = 20.9, *p* < .001, d = .83, Bootstrapped: M = 195.35, 98% CI (178, 212.8)].

The main effects were qualified by a significant two-way interaction between Label format and Category [F(6, 1110) = 3.6, *p* = .001, MSE = 44072.57, η_p_^2^ = .02]. Bonferroni’s post-hoc tests confirmed the pattern found for the Category main effect, that is slower RTs for proper weapons, faster for garments, with improper weapons in the middle, for all label formats (mean differences, pairwise comparisons test statistics, effect sizes, Bootstrapped means and CI for Bonferroni’s post-hoc tests for the two-way interaction between Label format and Category are reported in S2 Table).

Bonferroni’s post-hoc tests showed that both proper weapons and garments were detected significantly faster after an AV label than after any other label (all *p*s < .02).

**Fig 3:**
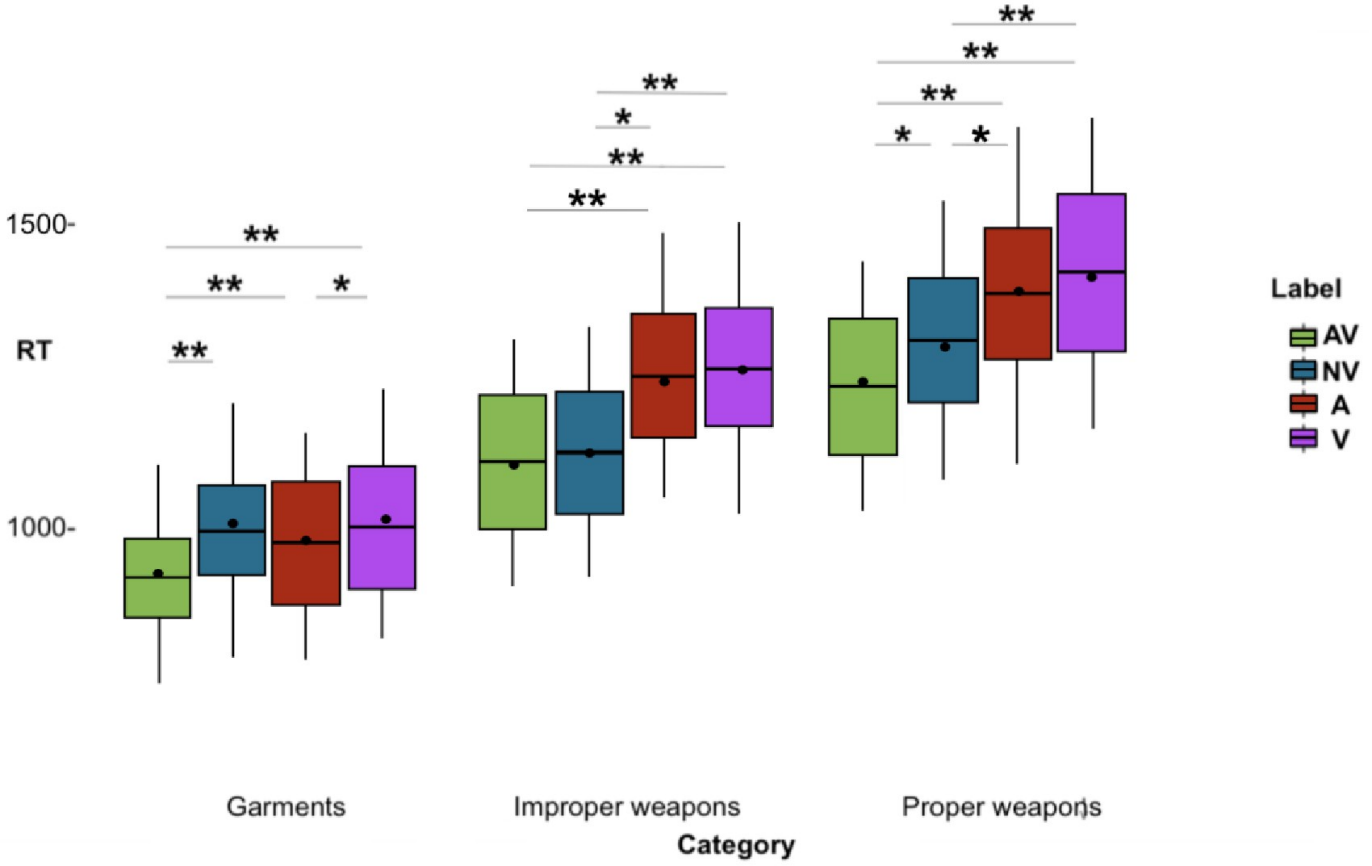
RTs (y-axis) for each category following the label formats (x-axis) are shown. AV (green box) = audio + visual; NV (blue box) = noise + visual, A (red box) = audio, V (purple box) = visual. The significance of the pairwise comparisons between label formats (Bonferroni’s post-hoc tests) is indicated by asterisks: ** (p < .001), *(p < .05). The box’s range, including whiskers, corresponds to the mean +/-3*SD, the thick line running the width of the box represents the median, the point inside the box represents the mean.

Improper weapons were detected significantly faster after an AV label and a NV label with respect to the A (*p*< .001 and *p* = .004, respectively) and V formats (all *p*s < .001). All mean differences, pairwise comparison test statistics, effect sizes, Bootstrapped means and CI are reported in Table 2 (see also Fig 3).

**Table 2:** Results of paired-comparisons between label and category.

**Pairwise comparisons**	**Mean difference**	**Test statistics (t)**	**Adjusted p-value**	**Cohen’s *d***	**Bootstrapped mean**	**Bootstrapped CI**
**Garments:**	**-82.75**	**t(185) = -6.21**	***p* < .001**	***d*=.46**	**-82.74**	**-104.8, -44.35**
**AV vs NV**						
**Garments:**	**-74.76**	**t(185) = -5.27**	***p* < .001**	***d*=.39**	**-74.98**	**-102.3, -48.6**
**AV vs A**						
**Garments:**	**-130.00**	**t(185) = -8.83**	***p* < .001**	***d*=.64**	**-129.76**	**-157.4, -101.6**
**AV vs V**						
Garments:	7.99	t(185) = 0.52	*p =* 1	*d*=.05	7.93	-21.91, 35.21
NV vs A						
Garments:	-47.24	t(185) = -3.12	*p =* .06	*d*=.23	-47.35	-81.05, -16.38
NV vs V						
**Garments:**	**-55.24**	**t(185) = -3.41**	***p =* .018**	***d*=.25**	**-55.23**	**-89.03, -23.79**
**A vs V**						
Improper weapons:	-28.65	t(185) = -1.84	*p =* 1	*d*=.13	-28.69	-59.58, -0.54
AV vs NV						
**Improper weapons**	-**93.75**	**t(185) = -6.06**	***p* < .001**	***d*=.44**	-**93.95**	-**123.1, -64.5**
**AV vs A**						
**Improper weapons:**	**-140.68**	**t(185) = -8.87**	***p* < .001**	***d*=.66**	**-141.17**	**-176.7, -106.1**
**AV vs V**						
**Improper weapons:**	**-65.11**	**t(185) = -3.8**	***p =* .004**	***d*=.28**	**-65.07**	**-98.67, -32.62**
**NV vs A**						
**Improper weapons:**	**-112.03**	**t(185) = -6.46**	***p* < .001**	***d*=.47**	**-111.60**	**-146.1, -78.34**
**NV vs V**						
Improper weapons:	-46.92	t(185) = -2.69	*p =* .21	*d*=.19	-46.86	-81.5, -11.07
A vs V						
**Proper weapons:**	**-54.15**	**t(185) = -3.41**	***p =* .02**	***d*=.25**	**-54.39**	**-85.78, -22.56**
**AV vs NV**						
**Proper weapons**	-**126.67**	**t(185) = -6.54**	***p* < .001**	***d*=.48**	-**125.87**	-**164.1, -87.22**
**AV vs A**						
**Proper weapons:**	**-164.64**	**t(185) = -9.12**	***p* < .001**	***d*=.67**	**-164.74**	**-204.3, -124.9**
**AVvs V**						
**Proper weapons:**	**-72.52**	**t(185) = -3.64**	***p =* .008**	***d*=.27**	**-72.44**	**-114.2, -30.87**
**NV vs A**						
**Proper weapons:**	**-110.49**	**t(185) = -5.93**	***p* < .001**	***d*=.44**	**-110.26**	**-151.1, -69.9**
**NVvs V**						
Proper weapons:	-37.96	t(185) = -1.75	*p =* 1	*d*=.13	-37.98	-85.99, 10.09
A vs V						

#### 3.2.2 Dprime

**Fig 4:**
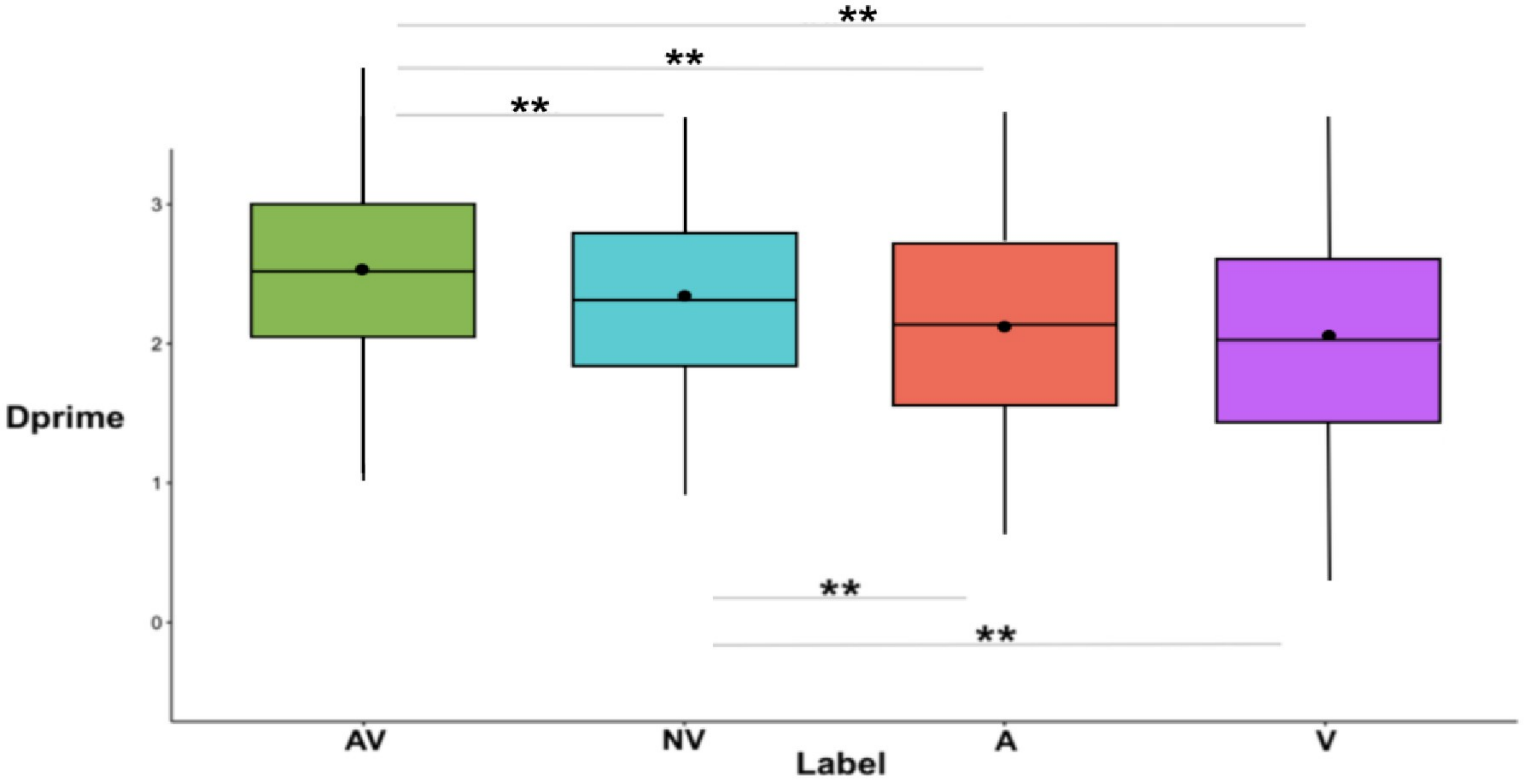
Dprime (y-axis) following each of the four label formats (x-axis) are shown. AV = audio + visual; NV = noise + visual, A = audio, V = visual. The significance of pairwise comparisons between label formats (Bonferroni’s post hoc tests) is indicated by asterisks: ** (*p* < .001).

The 4 (Label format) × 3 (Category) repeated measures ANOVA revealed main effects of Label format [F(3, 555) = 48.08, MSE = -0.04, *p* < .001, η_p_^2^ = .21] and Category [F(2, 370) = 185.56, *p* < .001, MSE = 0.01, η_p_^2^ = .50]. Bonferroni’s post-hoc tests revealed that dprime was higher after an AV label than after a NV label [t(557) = 4.5, *p* < .001, d = .23, Bootstrapped: M = 0.16, 98% CI (0.08, 0.24)], an A label [t(557) = 8.88, *p* < .001, d = .53, Bootstrapped: M = 0.37, 98% CI (0.28, 0.47)], and a V label [t(557) = 10.02, *p* < .001, d = .59, Bootstrapped: M = 0.40, 98% CI (0.30, 0.49)]. Similarly, dprime was higher after a NV label than after an A [t(557) = 5.27, *p* < .001, d = .23, Bootstrapped: M = 0.21, 98% CI (0.12, 0.30)], and a V label [t(557) = 6.11, *p* < .001, d = .35, Bootstrapped: M = 0.24, 98% CI (0.15, 0.33)] (see Table 3). No significant differences were found in dprime between A and V labels [t(557) = 0.80, *p* = 1, d = .04, Bootstrapped: M = 0.03, 98% CI (-0.06, 0.12)] (see Fig 4).

**Table 3:** Dprime for each condition of label format are reported below.

**Label format**	**dprime (sd)**
Audio + visual (AV)	2.50 (0.68)
Noise + visual (NV)	2.34 (0.70)
Audio (A)	2.13 (0.72)
Visual (V)	2.10 (0.68)

Moreover, dprime values were lower for improper weapons compared to proper weapons [t(1425.8) = -3.53, *p* < .001, d = .16, Bootstrapped: M = -0.13, 98% CI (-0.17, -0.08)] and garments [t(1392.1) = -16.22, *p* < .001, d = .74, Bootstrapped: M = -0.45, 98% CI (-0.63, -0.53)]. Dprime values for proper weapons were lower than those for garments [t(557) = -14.24, *p* < .001, d = .71, Bootstrapped: M = -0.45, 98% CI (-0.50, -0.40)].

The main effects were qualified by a significant two-way interaction between Label format and Category [F(6, 1110) = 3.39, *p* = .002, MSE = -0.015, η_p_^2^ = .02].

Bonferroni’s post-hoc tests confirmed the pattern found for the Category main effect, that is lower dprime values for improper weapons, higher for garments, with proper weapons in the middle, for A and V label formats. In AV and NV label formats, dprime values were still significantly higher for garments than for improper and proper weapons, but no significant difference was found between improper and proper weapons, though dprime values were lower for improper than for proper weapons (mean differences, pairwise comparisons test statistics, effect sizes, Bootstrapped means and CI for Bonferroni’s post-hoc tests for the two-way interaction between Label format and Category are reported in S2 Table).

**Fig 5:**
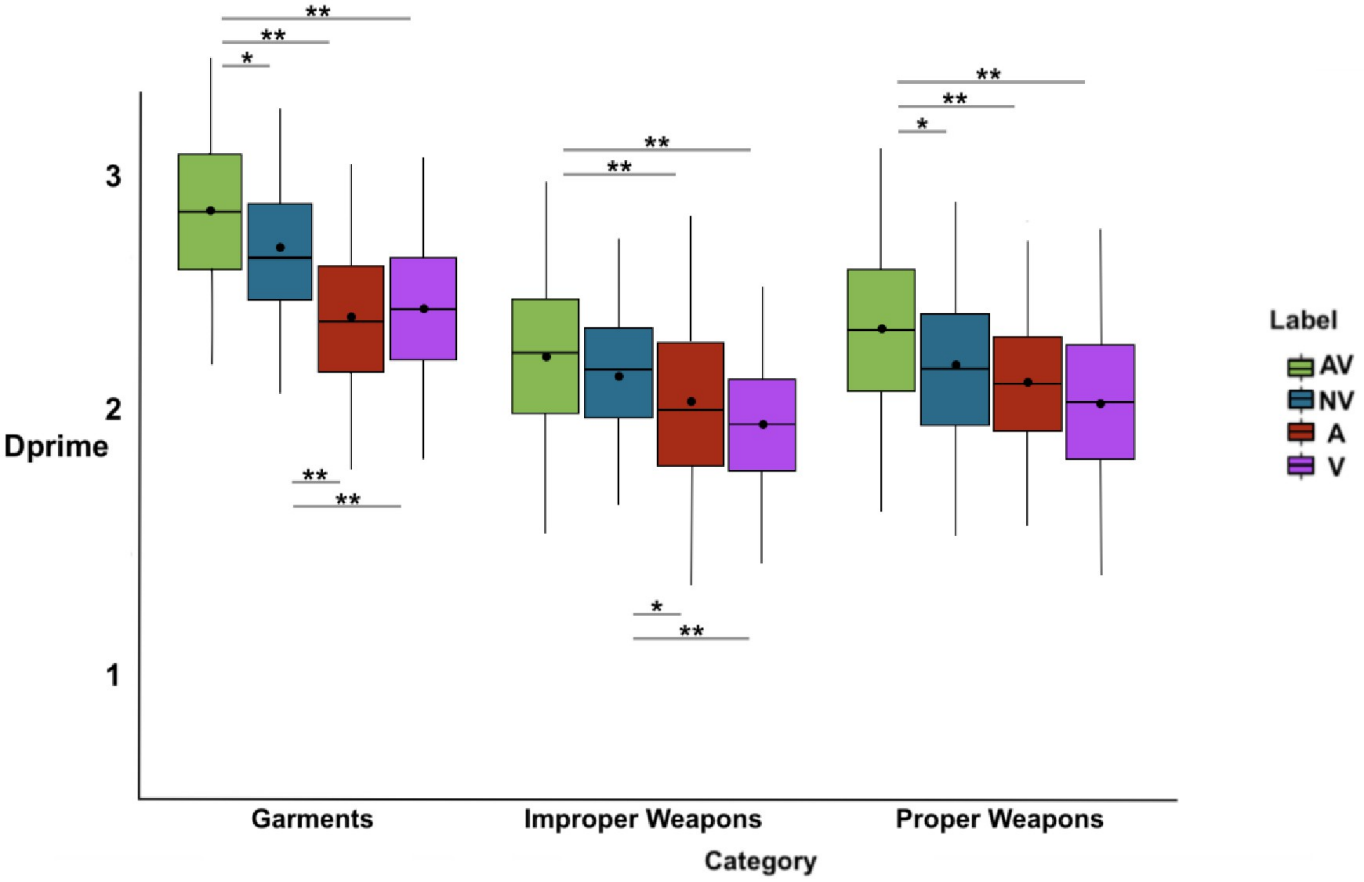
Dprime (y-axis) for each category following the label formats (x-axis) are shown. The significance of the pairwise comparisons between label formats (Bonferroni’s post-hoc tests) is indicated by asterisks: ** (p < .001), *(p < .05). The box’s range, including whiskers, corresponds to the mean +/-3*SD, the thick line running the width of the box represents the median, the point inside the box represents the mean.

Bonferroni’s post-hoc tests revealed that dprime was significantly higher after an AV compared to the NV (*ps* ≤ .01), A, V (both *p*s < .001) labels both for garments and for proper weapons, whereas dprime for improper weapons was higher after an AV and NV (*p* = 1) compared to an A (*p* < .001 and *p* = .01, respectively) and V label (both *ps* < .001). Mean differences, pairwise comparisons test statistic, effect sizes, Bootstrapped means and CI are in Table 4 (see also Fig 5) (for an analogous analysis on criterion see S2 File).

**Table 4:** Results of Bonferroni’s post-hoc paired-comparisons between label format and category for dprime, are shown in the table below.

**Pairwise comparisons**	**Mean difference**	**Test statistics (t)**	**Adjusted p-value**	**Cohen’s *d***	**Bootstrapped mean**	**Bootstrapped CI**
**Dprime_Garments:**	**0.19**	**t(185) = 3.66**	***p =* .006**	***d*=.32**	**0.18**	**0.06, 0.30**
**AV vs NV**						
**Dprime_Garments:**	**0.50**	**t(185) = 9.44**	***p* < .001**	***d*=.86**	**0.50**	**0.38, 0.62**
**AV vs A**						
**Dprime_Garments:**	**0.40**	**t(185) =7.91**	***p* < .001**	***d*=.77**	**0.40**	**0.28, 0.52**
**AV vs V**						
**Dprime_Garments:**	**0.31**	**t(185) = -5.56**	***p* < .001**	***d*=.52**	**0.31**	**0.18, 0.44**
**NV vs A**						
**Dprime_Garments:**	**-0.31**	**t(185) = 4.85**	***p* < .001**	***d*=.40**	**-0.31**	**-0.52, -0.11**
**NV vs V**						
Dprime_Garments:	-0.09	t(185) = -2.11	*p =* .65	*d*=.17	-0.09	-0.19, 0.00
A vs V						
Dprime_Improper weapons:	0.09	t(185) = 1.4	*p =* 1	*d*=.13	0.09	-0.06, 0.24
AV vs NV						
**Dprime_Improper weapons:**	**0.35**	**t(185) = 4.86**	***p* < .001**	***d*=.47**	**0.35**	**0.19, 0.53**
**AV vs A**						
**Dprime_Improper weapons:**	**0.45**	**t(185) = 7.03**	***p* < .001**	***d*=.65**	**0.45**	**0.31, 0.60**
**AV vs V**						
**Dprime_Improper weapons:**	**0.26**	**t(185) = 3.46**	***p =* .01**	***d*=.32**	**0.26**	**0.09, 0.44**
**NV vs A**						
**Dprime_Improper weapons:**	**0.36**	**t(185) = 5.45**	***p* < .001**	***d*=.47**	**0.35**	**0.20, 0.49**
**NV vs V**						
Dprime_Improper weapons:	0.09	t(185) = 1.32	*p =* 1	*d*=.12	0.09	-0.08, 0.26
A vs V						
**Dprime_Proper weapons:**	**0.20**	**t(185) = 3.44**	***p =* .01**	***d*=.32**	**0.20**	**0.06, 0.34**
**AV vs NV**						
**Dprime_Proper weapons:**	**0.26**	**t(185) = 4.64**	***p* < .001**	***d*=.41**	**0.26**	**0.13, 0.39**
**AV vs A**						
**Dprime_Proper weapons:**	**0.35**	**t(185) = 5.93**	***p* < .001**	***d*=.55**	**0.35**	**0.21, 0.48**
**AV vs V**						
Dprime_Proper weapons:	0.06	t(185) = 1.05	*p =* 1	*d*=.09	0.06	-0.07, 0.18
NV vs A						
Dprime_Proper weapons:	0.15	t(185) = 2.88	*p =* .07	*d*=.25	0.15	0.03, 0.27
NV vs V						
Dprime_Proper weapons:	0.09	t(185) = 1.81	*p =* 1	*d*=.14	0.09	-0.02, 0.21
A vs V						

### 3.3 Trait anxiety

A significant positive correlation was detected between participants’ scores at STAI-Y (Y2; 20 items, Cronbach’s α = .95, 95% CI (0.91, 0.98); M_Stai-y_ = 47.17, sd_Stai-y_ = 5.09 for the current sample) and mean RTs for correct responses in each category, both with the target present (proper weapons, r(184) = .49, *p* < .001; improper weapons, r(184) = .44, *p* < .001; garments, r(184) = .29, *p* < .001), and absent (proper weapons, r(184) = .35, *p* < .001; improper weapons, r(184) = .37, *p* < .001; garments, r(184) = .36, *p* < .001).

In the presence of the target, the correlation was significantly stronger with proper weapons and improper weapons than with garments (z = 5.09, *p* < .001 and z = 4.78, *p* < .001, respectively), whereas no significant difference between correlation coefficients for proper and improper weapons was found (z = 1.01, *p* = .17). Notably, when taking into account only the initial trials of each block in which garments were presented as the first category, no significant correlation was found between participants’ scores at STAI-Y and mean RTs for correct responses in target-present trials for the garments category (r(184) = .048, *p* = .67).

Since this result is based on a limited number of trials—no more than five for 163 participants, giving a total of 510 observations—as a further check we conducted the analysis also on the two categories of weaponry. In both cases, the correlation between participants’ scores at STAI-Y and mean RTs for correct responses in target-present trials remains (for proper weapons, r(157) = .24, p = .003; for improper weapons r(149) = .22, p = .004).

In the absence of the target, the correlation coefficients across categories did not differ (all *p*s > .006).

### 3.4 Summary of results

To recap, the main findings can be summarised as follows:

a. The preregistered test confirmed that correct RTs were faster in the AV label condition than in the NV label condition.
b. The AV advantage over NV was driven by target present trials, which were further analysed.
c. In each label format, proper weapons were detected significantly more slowly than improper weapons and garments, and improper weapons more slowly than garments.
d. Proper weapons and garments were detected significantly faster after an AV label than a NV label. No difference was found between AV and NV labels for improper weapons.
e. For proper and improper weapons, RTs after NV were faster than those after both A and V, and RTs after an A and V labels did not differ. For garments, RTs in the NV condition did not differ from any of the two unimodal conditions, and RTs after A were faster than RTs following V.
f. Dprime for garments and proper weapons was significantly higher after AV compared to NV and both the unimodal conditions, but not for improper weapons, for which AV and NV did not differ.
g. Dprime values were lower for improper weapons, higher for garments, with proper weapons in the middle, for A and V label formats. In both AV and NV, dprime values were still higher for garments, while improper and proper weapons did not differ from each other.
h. A significant positive correlation was detected between participants’ trait anxiety scores and mean RTs for correct responses for each category. With the target present, both the correlation coefficient of proper weapons and the correlation coefficient of improper weapons were significantly higher than that of garments; no difference was found between correlation coefficients of proper and improper weapons instead. When garments were presented as the first category, there was no correlation between trait anxiety scores and mean RTs for correct responses. Nonetheless, the correlation persists in both cases when improper and proper weaponry appears as the first category.

## 4. Discussion

In a preregistered study, we tested the label-feedback hypothesis against a matched multimodal condition. The main experimental and control conditions were the audio-visual (AV) and the white noise-visual (NV) label format, respectively, with the unimodal A and V label formats added to evaluate possible differences between multi- and unimodal conditions. The name of a target superordinate category, rather than the name of the target object, was used throughout as a cue and was presented immediately before the onset of a search grid. The use of superordinate category labels may allow to determine whether they provide enough linguistic expectations to be useful in the formulation of perceptual hypotheses on the incoming visual input and assess whether the superordinate label-feedback effect may be modulated by category type, with relevant implications for real-world searches. Following two prior investigations on the categorical status of improper weapons [48, 49], we chose proper weapons, improper weapons and garments as superordinate categories, with the extra purpose to gather further information on the processing of improper weapons as an ad-hoc category, by identifying similarities and/or differences with the structured category of garments on the one hand and the emotionally-loaded [48, 55] category of weapons on the other hand.

Results confirmed the preregistered prediction: There was an advantage of the AV label with respect to the NV label in RTs. This advantage was present and significant across all trials as shown by our preregistered analysis. An exploratory analysis on dprime confirmed the presence of an AV advantage over NV in a measure of sensitivity.

The auditory label in the AV condition may thus provide semantic information that is not fully redundant or engages in a dynamic interaction with the visual label. This is consistent with the idea that the AV condition prompted an “up regulation” of the label, a requirement for enhancing the label’s beneficial effects [15, 16, 17]. It also implies that, in addition to the basic level labels currently in use, superordinate categories are capable of producing a feedback effect. According to the label-feedback hypothesis [16], the activation of a word label (e.g. “hat”) affects the temporary representation and perception of the concept rather than merely providing access to it. The top-down activation of features induced by a word label would result in a transitory “perceptual warping” in which members of the same basic-level category are “dragged” further away from non-members (e.g. *plane*, *pencil* etc. but also other garments, such as *socks*, *trainers*, etc.) (i.e. inducing a temporarily warped categorization) [16]. By generating such a category expectation within which the incoming perceptual input is processed, the word label may exert an effect due to its assisting the formulation of hypotheses on the incoming visual input. The concept of labels eliciting categorical expectations can be expressed in terms of “categorical attention” [24, 88–90].

Further exploratory analyses showed that target-present trials were the main source of this effect. This suggests that the AV label may have primarily acted by “prioritizing” potential objects falling within the target category rather than facilitating distractor rejection.

In target-present trials, multimodal labels were typically associated to faster responses than unimodal labels, and within the latter, visual labels produced the slowest response. The multimodal advantage fits well with earlier findings in the literature on multimodal facilitation [91, 92]. Multimodal presentation involves an interaction between information in one sensory modality and information conveyed via another modality [93], with multisensory integration creating a more robust and reliable perceptual representation of the incoming stimulus. For instance, regardless of spatial congruency, temporally congruent auditory inputs can make a visual stimulus more salient, enhancing target visibility and detection [94–96].

The pattern (RTs_AV_ < RTs_NV_) < (RTs_A_ < RTs_V_) was generally respected, although a few differences were revealed: improper weapons differed from the other two categories in that this category showed AV-NV equivalence, and garments differed from the other two categories in that NV did not differ from both A and V (NV was in between the two). These latter significantly differed from each other only in garments, and more research will be required to provide precise explanations of how unimodal labels interact with the characteristics of a category.

At first glance, this pattern shows at least two aspects. First, the multimodal facilitation effect is not wholly accounted for by semantics, as the NV condition improves RTs with respect to the unimodal conditions in the two weapons categories, acting as a trigger. This finding fits well with recent proposals that consider attention orientation as a multi-sensory construction [97] rather than a primarily visual process [98], a consideration supported by neurophysiological investigations [99, 100]. Secondly, the influence of white noise may be conditioned by the level of familiarity. The high familiarity of garments could make the task less demanding in general (see the moderate brain arousal model, MBA) [76], preventing white noise from having a noticeable triggering effect. This issue merits further investigation as familiarity may be a variable affecting both the establishment of a white noise effect and the label-feedback process. Indeed, in a previous study [17] search speed was enhanced for more familiar compared to less familiar items, but there is no study that directly investigate the link between familiarity and white noise effects.

The finding that the multimodal superordinate categorical label can facilitate visual search for category members may lead to a better specification of the mechanism(s) involved in such a process. Lupyan [16] makes the claim that "names (verbal labels) play an active role in perception and categorization by selectively activated perceptual features that are diagnostic of the category being labeled” (p. 4). However, unlike basic categories, superordinate categories are characterized by lower visual similarity among the category members, although it has been shown that visual similarity plays a role in categorization [34].

The information provided by the superordinate label may thus activate objects “in a particular state” [88], just as labels at basic level have been reported to do [16]. According to the “instantiation principle” [101, 102] when a superordinate level concept is activated, information on its exemplars is activated as well. Murphy & Smith [103] have found that the activation of superordinate level concepts also triggers the activation of several exemplars of the category characterized by perceptual information, conveying both general knowledge [104] and functional information [105].

It may thus be the case that what is activated and triggers categorical attention and expectations are affordances [26, 35] rather than specific visual features of category members. Such primary affordances, e.g. being able to be grasped and/or worn, having an elongated shape, not be rigid enough so as to be bent, or a certain ratio of length to thickness, etc., may directly affect typical category member sharing affordances to a high degree. They may also operate indirectly via the activation of the motor programs associated with using these objects [106, 107] by processing words that denote manipulable objects [108, 109]. According to “*vision*-*for*-*action*” [110] or “*mental simulation”* [111] theories, simply hearing a word related to a graspable object or seeing the object itself, even without an intent to use it, activates sensorimotor areas of the brain associated with the actual potential object manipulation [112], thus guiding visual attention [113] and possibly favoring visual detection.

This account would nicely explain the lack of a label feed-back effect for improper weapons, shown by both our RTs and dprime data, as a result of the ambiguity between the primary, tool-linked affordances elicited by the label and the secondary, weapons-linked affordances, which are those that allow the categorization of these objects as a kind of weapon. The different patterning of AV vs NV between improper weapons and both proper weapons and garments supports the idea that these are distinct categories [48, 49], and it cannot be attributed to generic differences in absolute RTs. Indeed, absolute RTs to improper weapons were positioned in-between those to garments (the fastest) and those to proper weapons (the slowest).

The fact that the label-feedback effect induced by a superordinate label may be affected and modulated by the inherent characteristics of the category merits further investigation to ascertain whether the characteristics of the category also influence the label-feedback effect when a basic or a subordinate label is used.

Another notable finding concerns the relation between trait anxiety and search performance. Given that the search targets of our experiment include weapons, simply processing these items may arouse anxiety, increasing engagement with such stimuli or inhibiting disengament [54, 58, 114]. Indeed, two results seem to confirm that feelings associated with threats [50–52], although co-varying with familiarity, did affect RTs in the task of visual search. First, regardless of whether the target was present or not, participants with higher trait anxiety scores (STAI-Y2) tended to be slower overall. Second, a significant positive correlation was detected between participants’ trait anxiety scores (STAI-Y2) and mean RTs for correct responses for each category, though the correlation for garments disappeared when these were presented as the first category of the task (while it persisted for both proper and improper weapons). This means that responses for garments were slower when they were not presented as the first category and it may imply that having previously encountered instances of weapons in the study could have slowed down also the responses to garments [115, 116], as a sort of contextual effect.

A likely explanation is that of an interaction between attentional bias and the generally slower processing efficiency found to be associated with high trait anxiety [51]. The attentional control theory (ACT) [51] proposes that trait anxiety disrupts functional efficiency of executive control of attention relating to working memory system such as shifting. The greater slowness of anxious people with weapons could stem from the fact the high level of anxiety involves a greater analysis of the items before responding [117]. This could explain because anxious people are slower also in target-absent trials [118, 119].

In conclusion, the AV categorial label we adopted differs from the format typically used in studies on the label-feedback hypothesis (involving the repetition aloud of a visual label at basic category level) [17, 23]. Besides, the superordinate label, which was never employed in previous experiments on the label-feedback effect in visual search tasks, has demonstrated its capacity to aid visual search. Although this study offers promising evidence, more research is required to determine the precise importance of the intrinsic qualities of categories (i.e. familiarity) in the label feedback effect.

As a final note, the discovery that different formats of a label presented in conjunction with an object modulate the perception of the object and that such modulation is related to the type of category may be exploited for application to real-word situations. One such situation is X-ray baggage screening, the practice of visually checking X-ray images of baggage for threats and forbidden goods, a crucial component of airport security, that is open to effects of search strategies [120]. The use of an AV categorial label might be appropriate in this context (i.e. by providing essential information about the target category, without prompting fixation on a specific object), but more research is needed to determine whether the label feedback effect extends and can be usefully applied to more complex real-world situations, where multiple target categories are being searched for at once.

## Supporting information

S1 Table The value of imagery concordance, familiarity and subjective visual complexity of the various items. (PDF)

S2 Table Results of paired-comparisons between Label format and category for RTs and dprime. (PDF)

S1 File The values of the indices of visual complexity for each trial. (XLSX)

S2 File The analyses on criterion. (PDF)
