# Supplementary material for: The label-feedback effect is influenced by target category in visual search
Source: PLoS One. 2024 Aug 1;19(8):e0306736. doi: 10.1371/journal.pone.0306736 (PMC11293709; doi:10.1371/journal.pone.0306736)
Supplement: S1 Table — (PDF) [file pone.0306736.s001.pdf]

S1 Table. In the table below, the value of imagery concordance, familiarity and subjective visual complexity of the various items are listed for the three categories.

**Items: Proper weapons**	**Imagery concordance**	**Familiarity**	**Visual complexity**
undershirt	4.27	4.77	2.21
jeans	4.32	4.2	2.87
sneaker	4.05	4.73	3.41
shirt	4.43	4.91	2.62
sock	4.23	4.35	2.11
hat	2.66	3.69	2.45
jacket	3.27	3.05	2.77
cap	3.66	3.93	2.19
skirt	3.37	3.74	2.78
heel shoe	3.12	3.83	3.45
**Improper**			
baseball bat	3.52	3.55	2
cutter	3.82	3.67	2.98
screwdriver	3.77	3.66	2.56
hammer	3.89	3.51	3.11
scissors	3.99	4.45	2.82
cooking [illegible]	3.33	2.89	2.79
tube	3.34	2.93	1.58
razor	2.70	3.97	3.44
blowpipe	2.81	2.96	3.32
pliers	3.37	3.31	3.01
**Proper weapons**			
puncher	3.99	2.70	3.11
dynamite	3.85	2.88	2.66
gun	3.83	2.97	2.82

bullets	3.75	3.03	2.67
bomb	4.01	2.53	2.69
rifle	3.66	2.43	2.69
flare gun	3.29	2.14	2.54
dagger	3.21	2.58	3.27
baton	3.06	2.3	2.04
detonator	2.82	2.16	2.86
