# Supplementary material for: The label-feedback effect is influenced by target category in visual search
Source: PLoS One. 2024 Aug 1;19(8):e0306736. doi: 10.1371/journal.pone.0306736 (PMC11293709; doi:10.1371/journal.pone.0306736)
Supplement: S2 Table — (PDF) [file pone.0306736.s002.pdf]

S2 Table. Results of paired-comparisons between Label format and Category for RTs and dprime, are shown in the below tables.

Pairwise comparisons for RTs	Mean difference (ms)	Test statistics (t)	Adjusted p-value	Cohen's *d*	Bootstrapped mean	Bootstrapped CI
AV: Garments vs Improper weapons	-201.62	t(738.28) = -14.81	*p* < .001	*d*= 1.11	-201.38	-227.5, -176.6
AV: Garments vs Proper weapons	-270.32	t(718.94) = -19.35	*p* < .001	*d*= 1.42	-270.50	-297.4, -245.4
AV: Improper weapons vs Proper weapons	-68.70	t(735.87) = -4.73	*p* < .001	*d*= .35	-68.75	-95.84, -41.74
NV: Garments vs Improper weapons	-131.03	t(731.11) = -7.79	*p* < .001	*d*=.58	-131.64	-161.4, -103.9
NV: Garments vs Proper weapons	-205.61	t(739.77) = -12.68	*p* < .001	*d*=.93	-205.72	-238.9, -170.5
NV: Improper weapons vs Proper weapons	-74.58	t(738.63) = -4.32	*p* < .001	*d*=.32	-74.81	-105.7, -43.19
A: Garments vs Improper weapons	-220.61	t(736.21) = -13.52	*p* < .001	*d*= .99	-220.53	-252.3, -187.4
A: Garments vs Proper weapons	-321.96	t(671.58) =-16.99	*p* < .001	*d*=1.24	-321.76	-362.2, -283.3
A: Improper weapons vs Proper weapons	-101.34	t(700.93) = -5.18	*p* < .001	*d*=.38	-101.66	-139.2, -65.49
V: Garments vs Improper weapons	-212.30	t(738.31) = -12.38	*p* < .001	*d*=.91	-212.57	-250.2, -176.7
V: Garments vs Proper weapons	-304.96	t(708.82) = -16.33	*p* < .001	*d*=1.2	-304.98	-344.4, -266
V: Improper weapons vs Proper weapons	-92.66	t(726.11) = -4.82	*p* < .001	*d*=.35	-93.01	-132.1, -55.9

Pairwise comparisons for dprime	Mean difference	Test statistics (t)	Adjusted p-value	Cohen's *d*	Bootstrapped mean	Bootstrapped CI
**AV: Garments vs Improper weapons**	**0.63**	**t(360.51) = 10.001**	***p* < .001**	***d*= .86**	**0.63**	**0.44, 0.92**
**AV: Garments vs Proper weapons**	**0.52**	**t(361.22) = 8.32**	***p* < .001**	***d*= .64**	**0.521**	**0.23, 0.75**
AV: Improper weapons vs Proper weapons	-0.11	t(369.98) = -2.59	*p* = 1	*d*= .11	-0.108	-0.33, 0.11
**NV: Garments vs Improper weapons**	**0.58**	**t(344.11) = 7.46**	***p* < .001**	***d*= .58**	**0.58**	**0.34, 0.76**
**NV: Garments vs Proper weapons**	**0.51**	**t(369.63) = 8.90**	***p* < .001**	***d*= .65**	**0.518**	**0.36, 0.79**
NV: Improper weapons vs Proper weapons	-0.07	t(338.8) = -2.03	*p* = 1	*d*=. 06	-0.07	-0.24, 0.09
**A: Garments vs Improper weapons**	**0.48**	**t(335.7) = 6.41**	***p* < .001**	***d*= .52**	**0.485**	**0.23, 0.61**
**A: Garments vs Proper weapons**	**0.23**	**t(369.4) = 4.45**	***p* < .001**	***d*= .33**	**0.284**	**0.07, 0.49**
**A: Improper weapons vs Proper weapons**	**-0.25**	**t(342.4) = -4.9**	***p* = .008**	***d*= .36**	**-0.25**	**-0.44, -0.06**
**V: Garments vs Improper weapons**	**0.68**	**t(326.29) = 10.38**	***p* < .001**	***d*= .91**	**0.68**	**0.42, 0.9**
**V: Garments vs Proper weapons**	**0.41**	**t(353.9) = 5.7**	***p* < .001**	***d*= .44**	**0.41**	**0.25, 0.67**
**V: Improper weapons vs Proper weapons**	**-0.27**	**t(360.11) = -4.95**	***p* = .003**	***d*= .37**	**-0.27**	**-0.52, 0.1**
