# Supplementary material for: The label-feedback effect is influenced by target category in visual search
Source: PLoS One. 2024 Aug 1;19(8):e0306736. doi: 10.1371/journal.pone.0306736 (PMC11293709; doi:10.1371/journal.pone.0306736)
Supplement: S2 File — (PDF) [file pone.0306736.s004.pdf]

In addition to dprime, we also investigated the possibility of a difference in criterion (a measure of response bias; see Verghese, 2001) between categories for the different label forms.

The 4 (Label format) × 3 (Category) repeated measures ANOVA revealed main effects of Label format [F(3, 555) = 22.54, MSE= 0.02, $p < .001$, $\eta_p^2 = .11$] and Category [F(2, 370) = 284.27, $p < .001$, MSE= 0.03, $\eta_p^2 = .60$]. Bonferroni's post-hoc tests revealed that criterion was lower after an AV label format than after a NV label [t(1105)= -3.34, $p < .001$, d = .20, Bootstrapped: M = -0.22, 98% CI (-0.34, -0.09)], an A label [t(1094)= -2.73, $p = .006$, d = .16, Bootstrapped: M = -0.18, 98% CI (-0.32, -0.05)], and a V label [t(1093)= -6.81, $p < .001$, d = .42, Bootstrapped: M = -0.46, 98% CI (-0.59, -0.32)]. Criterion did not differ between the NV and the A label formats [t(1111)= 0.50, $p = .6$, d = .02, Bootstrapped: M = 0.03, 98% CI (-0.09, 0.16)]; on the contrary, criterion was lower after NV in respect to the V label [t(1111)= -3.41, $p < .001$, d = .15, Bootstrapped: M = -0.24, 98% CI (-0.36, -0.10)] (see Table 3). Significant differences were found in criterion between A and V labels [t(1114)= -3.83, $p < .001$, d = .18, Bootstrapped: M = -0.27, 98% CI (-0.39, -0.14)].

Moreover, criterion was lower for improper weapons compared to proper weapons [t(1436)= - 15.47, $p < .001$, d = .81, Bootstrapped: M = -0.75, 98% CI (-0.86, -0.63)] and garments [t(1345)= -25.44, $p < .001$, d = 1.32, Bootstrapped: M = -1.35, 98% CI (-1.48, -1.23)]. Criterion for proper weapons was lower than for garments [t(1455)= -10.54, $p < .001$, d = .60, Bootstrapped: M = -0.60, 98% CI (-0.92, -0.47)].

The main effects were qualified by a significant two-way interaction between Label format and Category [F(6, 1110) = 10.6, $p < .001$, MSE= 0.87, $\eta_p^2 = .05$]. Bonferroni's post-hoc tests confirmed the pattern found for the Category main effect, that is lower criterion for improper weapons, higher for garments, with proper weapons in the middle, for each label formats (all *p*s ≤ .01) (mean differences, pairwise comparisons

test statistics, effect sizes, Bootstrapped means and CI for Bonferroni's post-hoc tests for the two-way interaction between Label format and Category are reported in the below S3 Table).

**Pairwise comparisons for Criterion**	**Mean difference**	**Test statistics (t)**	**Adjusted p- value**	**Cohen's *d***	**Bootstrapped mean**	**Bootstrapped CI**
**AV: Garments vs Improper weapons**	**0.92**	**t(185) = 8.92**	***p* < .001**	***d*= .91**	**0.92**	**0.67, 1.17**
**AV: Garments vs Proper weapons**	**0.53**	**t(185) = 5.7**	***p* < .001**	***d*= .59**	**0.52**	**0.31, 0.74**
**AV: Improper weapons vs Proper weapons**	**-0.39**	**t(185) = -3.7**	***p* < .001**	***d*= .45**	**-0.4**	**-0.62, -0.14**
**NV: Garments vs Improper weapons**	**1.37**	**t(185)= 13.1**	***p* < .001**	***d*= 1.26**	**1.37**	**1.15, 1.6**
**NV: Garments vs Proper weapons**	**0.84**	**t(185) = 8.8**	***p* < .001**	***d*= .87**	**0.84**	**0.63, 1.06**
**NV: Improper weapons vs Proper weapons**	**-0.53**	**t(185) = -4.82**	***p* < .001**	***d*= .51**	**-0.53**	**-0.77, -0.29**
**A: Garments vs Improper weapons**	**1.54**	**t(185) = 15.13**	***p* < .001**	***d*= 1.11**	**1.54**	**1.30, 1.77**
**A: Garments vs Proper weapons**	**0.46**	**t(185) = 4.7**	***p* < .001**	***d*= .51**	**0.45**	**0.22, 0.68**
**A: Improper weapons vs Proper weapons**	**-1.08**	**t(185) = -9.4**	***p* < .001**	***d*= .94**	**-1.07**	**-1.34, -0.83**
**V: Garments vs Improper weapons**	**1.57**	**t(185) = 14.6**	***p* < .001**	***d*= 1.13**	**1.56**	**1.31, 1.81**
**V: Garments vs Proper weapons**	**1.17**	**t(185) = 12.48**	***p* < .001**	***d*= 1**	**1.16**	**0.95, 1.4**
**V: Improper weapons vs Proper weapons**	**-0.40**	**t(185) = -3.61**	***p* < .001**	***d*= .46**	**-0.40**	**-0.66, -0.15**

Criterion, which with an impartial observer stabilizes on a value of about 1.0, represents an observer's bias to answer "yes" or "no." The perceived effects of a decision may influence the criterion that is used.

Criterion approaches 0.0 as the bias to say "yes" grows (liberal), leading to a higher hit-rate and false-alarm-rate. A respondent can be more inclined to indicate "Signal Present" if the costs of not detecting a signal while it is present are higher.

On an open-ended scale, criterion climbs over 1.0 as the inclination to say "no" increases (conservative), which lowers the hit-rate and false-alarm rate. Saying "Signal Present" when the signal is actually absent might have negative implications, and these would make the respondent less inclined to adopt a conservative criterion.

In our case, the participants displayed a criterion where improper and proper weapons were responded to less conservatively than garments, with proper weapons in the middle. This could indicate that in the case of potentially harmful object there may have been bias towards the affirmative response. This may be due to the fact that, in general, failing to locate a weapon could have more awful consequences than falsely reporting its presence.

For improper weapons, criterion reached lower values than for proper weapons, which suggests two considerations: first, that improper weapons were not treated as equivalent to proper weapons, and second, that participants adopted a particularly cautious approach with this kind of weaponry, which may be harder to identify due to its dual use.

Reference

Verghese, P. Visual search and attention: a signal detection theory approach. Neuron. 2001 Aug 30;31(4):523-35. doi: 10.1016/s0896-6273(01)00392-0. PMID: 11545712.)
